# Medical Image Fusion Based on Feature Extraction and Sparse Representation

**DOI:** 10.1155/2017/3020461

**Published:** 2017-02-21

**Authors:** Yin Fei, Gao Wei, Song Zongxi

**Affiliations:** ^1^Xi'an Institute of Optics and Precision Mechanics, Chinese Academic of Sciences, Xi'an 710119, China; ^2^University of Chinese Academy of Sciences, Beijing 100049, China

## Abstract

As a novel multiscale geometric analysis tool, sparse representation has shown many advantages over the conventional image representation methods. However, the standard sparse representation does not take intrinsic structure and its time complexity into consideration. In this paper, a new fusion mechanism for multimodal medical images based on sparse representation and decision map is proposed to deal with these problems simultaneously. Three decision maps are designed including structure information map (SM) and energy information map (EM) as well as structure and energy map (SEM) to make the results reserve more energy and edge information. SM contains the local structure feature captured by the Laplacian of a Gaussian (LOG) and EM contains the energy and energy distribution feature detected by the mean square deviation. The decision map is added to the normal sparse representation based method to improve the speed of the algorithm. Proposed approach also improves the quality of the fused results by enhancing the contrast and reserving more structure and energy information from the source images. The experiment results of 36 groups of CT/MR, MR-T1/MR-T2, and CT/PET images demonstrate that the method based on SR and SEM outperforms five state-of-the-art methods.

## 1. Introduction

Medical imaging attracts more and more attention due to the increasing requirements of clinic investigation and disease diagnosis. Owing to different imaging mechanisms, medical images of different modals provide a variety of complementary information about the human body in a limited domain. For example, the computed tomography (CT) images provide better information on dense tissue, the positron emission tomography (PET) images supply better information on blood flow and tumor activity with low space resolution, and the magnetic resonance (MR) images show better information on soft tissue. Moreover, the MR-T1 images give more detailed information about anatomical structures, whereas the MR-T2 images contain a greater contrast between the normal and abnormal tissues [1–4]. However, single multiple modality cannot satisfy the demand of images with high resolution and visualization for disease diagnosis.

In this regard, medical image fusion is a useful and powerful technique for integrating complementary information from multimodality images to improve the diagnostic accuracy. Besides, the fused images are more suitable for assisting the doctors in diagnosis and treatment planning [5]: fusing MR and CT images can generate the images which can describe the soft tissue and bone in order to concurrently represent anatomical and physiological features of the human body [6, 7]. MR-T1 and MR-T2 images are fused to segment white matter lesions and guide neurosurgical resection of epileptogenic lesions [7, 8]. In oncology, the combined PET/CT imaging is helpful to view the anatomical, physiological characteristics and the tumor activity [9, 10]. More than that, medical image fusion not only helps in diagnosing diseases but also reduces the storage cost [8].

As the most popular technique of the image fusion, the multiscale decomposition methods have developed quickly in recent years, such as discrete wavelet transform (DWT) [3, 7], framelet transform [9], contourlet transform [10], and nonsubsampled contourlet transform (NSCT) [1, 4, 6]. Unfortunately, transform-based methods produce poor fusion results in the presence of noise and it is difficult to choose the decomposition levels [11, 12].

Sparse representation (SR) has proven to be an exceedingly powerful tool for analyzing the signals of high dimensionality [13], so more and more researchers adapt SR to the field of image fusion for the purpose of getting better fused results [14–19]. However, the standard SR does not take the intrinsic structure [14] and the time complexity [17] into consideration. Therefore, adding them into the SR model is a reasonable strategy to improve the performance of SR [14], but it is complicated to find the relationship between the intrinsic structure information and the sparse coefficients. Reference [14] proposed a dictionary learning method combining with the geometrical structure by group sparse coding, but it did not talk about the time complexity of the algorithms based on sparse representation. Image fusion methods based on joint sparse representation (JSR) [11, 12, 20] need much more iterations to realize image vectors sparse representation for the trained dictionary with bigger size. Some researchers proposed some novel methods combining multiscale transform and SR [15, 18, 21], to put the structure information of the source images into the fused images, which make methods much more complex and time-consuming.

In that way, how to realize image fusion based on SR with local structure information in shorter time became the chief task. The decision map can help us achieve this goal by extracting the local structure feature of the image blocks [22–27]. Unfortunately, most of the methods combining with decision map are only appropriate for multifocus image fusion [22–25]. References [26, 27] apply the decision map into infrared and visible image fusion, which demonstrate that the decision map can be suitable for other type image fusion. In fact, for the methods based on SR, almost all the sparse coefficients fusion rules depend on different blocks feature values, which means they all belong to the methods based on decision map [28]. To realize the medical image fusion based on SR with decision map, we add the local structure and energy information of source images into the decision map to improve the speed of the algorithm and the quality of the fused results.

The main contribution of this paper is as follows:

1. To add the local structure and energy information of the source images into the SR algorithm for medical image fusion, we design three decision maps to extract the local energy and structure features of the source images.
2. It is good to use the decision to reduce the number of image blocks to sparse representation, so that we can get the results in much shorter time. Using the maps to remain more structure and energy information in fused images will also improve the quality of the results.

## 2. The Framework of the Proposed Method

**Figure 1:**
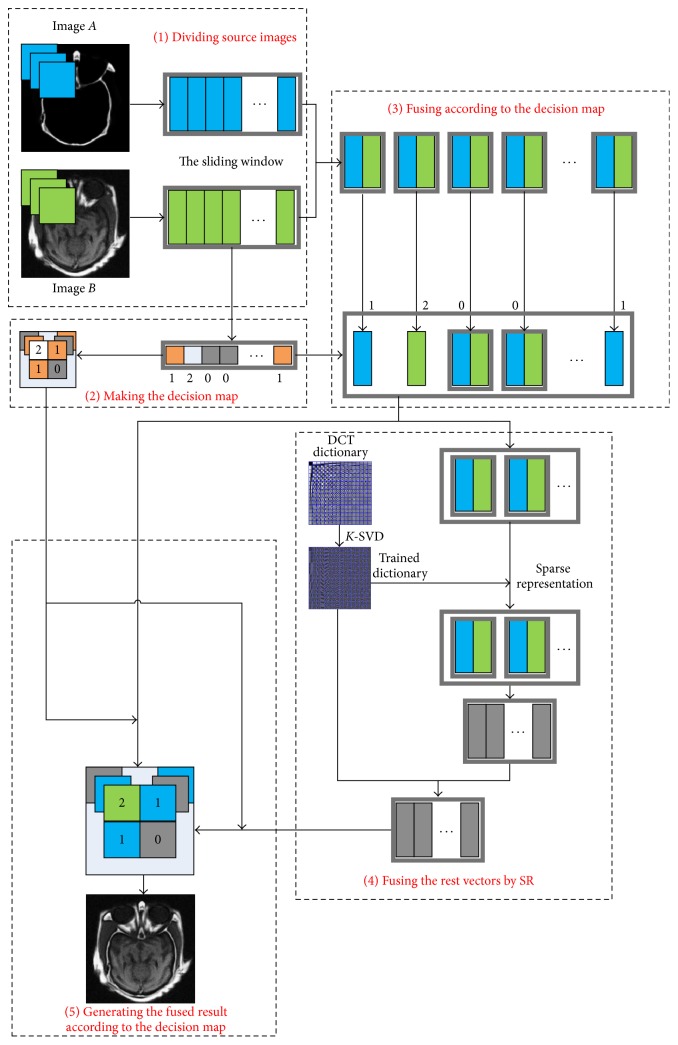
The framework of the image fusion method based on sparse representation and feature extraction.

There is the framework of the proposed approach based on SR and feature extraction as shown in Figure 1.

Firstly, we divide all source images *A* and *B* with the size of *m* × *n* into patches *y*_1_^*i*,*j*^ and *y*_2_^*i*,*j*^ through a sliding window with the size of ×*w*(*i* ≤ *m* − *w*, *j* ≤ *n* − *w*). All patches are arranged into vectors *V*_1_^(*i*−1)*∗*(*n*−*w*)+*j*^ and *V*_2_^(*i*−1)*∗*(*n*−*w*)+*j*^ from left to right and from top to bottom.

Secondly, we group these vectors into vector pairs according to corresponding positions of original patches and design the decision map according to their features separately.

Thirdly, we use the decision map to determine which one vector of each group as the result when the map is marked as 1 or 2. It means that these groups are regarded as the input of the sparse representation system when the map is marked 0.

Fourthly, we fuse the other vector pairs by the SR method.

Finally, the system can generate the fused results according to the decision map. The overlaps of the patches are averaged.

### 2.1. SR

In SR algorithms, a signal can be expressed as a sparse combination of the fewest possible atoms of an overcomplete dictionary [29]. Let *V* ∈ *R*^*m*^ denote a signal vector from the source images and let *D* ∈ *R*^*m*×*k*^ (*k* > *m*) denote an overcomplete dictionary whose column vectors are its atoms. The signal vector can be represented as *V* = *Dθ*, where *θ* ∈ *R*^*k*^ is a sparse coefficient vector. The sparse coefficient vector is acquired by solving the following question:

(1) θ^=arg minθ θ0s.t. V−Dθ22≤ε,

where  *ε*  is error tolerance parameter. An image vector can be represented as a superposition of the smallest possible number of atoms in the dictionary. We can solve (1) by OMP, BP, or other algorithms [12], because it is an NP-hard problem. It is important to find an appropriate dictionary for SR. There are two main approaches to build a dictionary. One is to use a fixed dictionary such as the Gabor dictionary [30], the discrete cosine transform (DCT) dictionary [31], and the Haar dictionary [32]. Another is to train a dictionary from a large number of training image patches, like* K*-SVD [32], which usually shows better performance in image processing compared to the fixed dictionary methods [11].

### 2.2. The Energy Map and the Structure Map

Let us regard ${\overset{-}{y}}_{1}^{i,j}$ and ${\overset{-}{y}}_{2}^{i,j}$ as mean values of *y*_1_^*i*,*j*^ and *y*_2_^*i*,*j*^, respectively. We use ‖*y*_1_^*i*,*j*^‖_2_ as the sign of energy and the mean square deviation $\left(1/64\right){\left\|y_{1}^{i,j}-{\overset{-}{y}}_{1}^{i,j}\right\|}_{2}$ as the sign of energy distribution for *y*_1_^*i*,*j*^, which are similar to *y*_2_^*i*,*j*^. We design the first decision map EM ∈ *R*^*M*×*N*^ (where *M* = *m* − *w* and *N* = *n* − *w*) which called the energy map by

(2) t1=164y1i,j2−y2i,j2,t2=164y1i,j−y−1i,j2−y2i,j−y−2i,j2,EMi,j=1,t1>0,  t2>0,2,t1<0,  t2<0,0,others.

In this way, our map contains the energy and energy distribution information of the vector pairs. However, this map does not contain enough image structure information. So we use the Laplacian of a Gaussian (LOG) to detect the structure information of the source images [33]. For noise cleaning, we smooth the image by convolving it with a digital mask corresponding to Gaussian function. The Gaussian function is expressed by (3)–(5) and we can get the local normalized structure information by (6). One has

**Figure 2:**
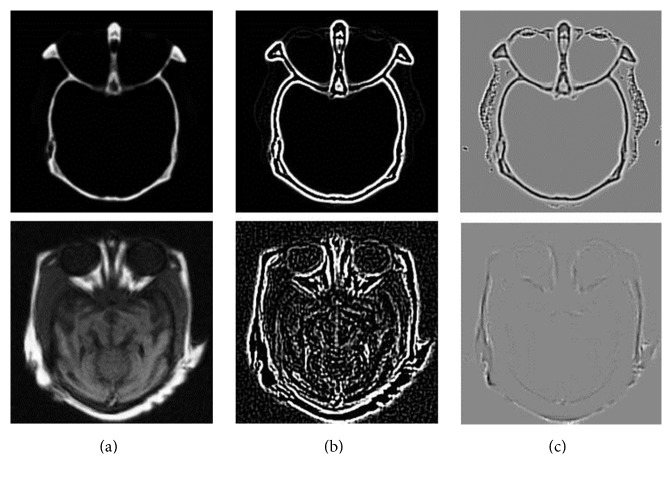
(a) The source images, (b) the structure information of LOG, and (c) the local structure information normalization.

(3) hx,yhr=−e−r2/2σ2,

(4) ∇2gr∗hrgr∗∇2hr,

(5) pgr∗∇2hr=−grr2−σ2σ4e−r2/2σ2,

(6) epmax⁡p,

where *r*^2^ = *x*^2^ + *y*^2^ and *σ* is the mean square deviation. Given an image matrix *g*(*x*, *y*), the LOG of the image function is the second-order partial derivatives along *x* and *y* directions. There is an example of the LOG edge detection of CT and MR images as shown in Figure 2.

*e*_1_^*i*,*j*^ and *e*_2_^*i*,*j*^ represent the local normalized structure information of *y*_1_^*i*,*j*^ and *y*_2_^*i*,*j*^, respectively. Therefore, we design the second map SM ∈ *R*^*M*×*N*^ named the structure map by

(7) s1=164e1i,j2−e2i,j2,s2=164e1i,j−e−1i,j2−e2i,j−e−2i,j2,SMi,j=1,s1>0.05,  s2>0,2,s1<−0.05,  s2<0,0,others.

### 2.3. The Structure and Energy Map

Combining the energy, energy distribution, and structure information, we design the third map SEM ∈ *R*^*M*×*N*^ which we name the structure and energy map by

(8) $$\begin{array}{c} SEM_{}^{i,j}=\left\{\begin{array}{cc} 0, & EM_{}^{i,j}=0,\;\;SM_{}^{i,j}=0,\\ 1, & EM_{}^{i,j}=1,\\ 1, & EM_{}^{i,j}=0,\;\;SM_{}^{i,j}=1,\\ 2, & EM_{}^{i,j}=2,\\ 2, & EM_{}^{i,j}=0,\;\;SM_{}^{i,j}=2. \end{array}\right. \end{array}$$

When *V*_1_^*h*^ = *Dθ*_1_^*h*^ and *V*_2_^*h*^ = *Dθ*_2_^*h*^, we can get the fusion vectors by (9) according to the decision map:

(9) gθ1h,θ2h=θ1h,θ1h>θ2h,θ2h,θ1h≤θ2h,Fh=V1h,Mi,j=1,V2h,Mi,j=2,D×gθ1h,θ2h,Mi,j=0,i=hn−w,  j=h−i∗n−w,

where *M* ∈ *R*^*M*×*N*^ can be EM, SM, SEM, or other decision maps.

In general, the proposed method at least has three merits in contrast to the normal SR based method. Firstly, it can make the fused results preserve the information of the source images as much as possible and remit the effect of algorithm noise. Secondly, it can get the results more rapidly because we just sparse-represent a part of the vector pairs. Thirdly, our algorithm combines energy, energy distribution, and structure characteristics of the images to enhance contrast of the results. In the abstract, the results fused by proposed have the best contrast information, which is the most important information to locate the position of the abnormal tissue.

## 3. Experiments

**Figure 3:**
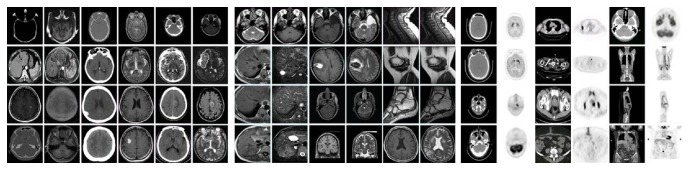
The source images for fusion and training dictionary, including 12 pairs of CT/MR images, 12 pairs of MR-T1/MR-T2 images, and 12 pairs of CT/PET images.

**Figure 4:**
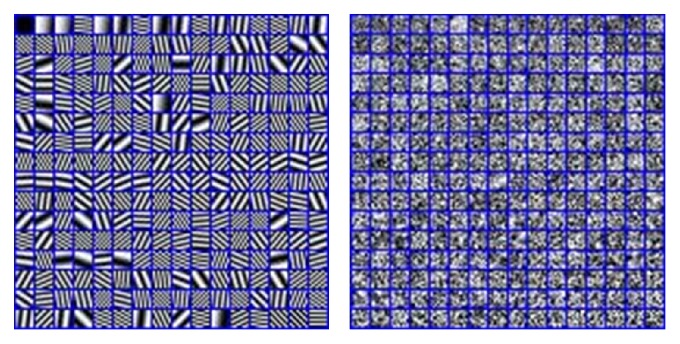
The DCT dictionary and the trained dictionary.

To evaluate the performance of the proposed method, three experiments are implemented. All the images are the same size of 256 × 256 pixels. In this paper, we train the dictionary with* K*-SVD using the pictures as shown in Figure 3. The error tolerance *ε* is set to be 0.01. The maximum iterations of the* K*-SVD are set to be 30. The initial dictionary is the DCT dictionary with the size of 64 × 256. We use OMP to estimate the sparse coefficients for simplicity. The moving step of the sliding window is set to be one pixel. We use three kinds of medical image pairs including CT/MR images, MR-T1/MR-T2 images, and CT/PET images to test the performances of those abovementioned methods. The DCT dictionary and trained dictionary are shown in Figure 4. The window size of LOG is set to be 5 × 5, and *σ* is set to be 2.

For comparison, five state-of-the-art methods are evaluated in the experiments, including methods based on NSCT [1, 6], method based on JSR [12], and methods based on NSCT and SR [15, 18]. In this paper, five objective evaluation measurements parameters are adopted to evaluate the fusion performance. There are local quality index (*Q*_0_) [34], weighted fusion quality index (*Q*_*W*_) [34], edge-dependent fusion quality index (*Q*_*E*_) [34], *Q*_*AB*/*F*_ [35] which measures the transmission of edge and visual information from source images to fused images, and mutual information (MI) [36] which computes the information transformed from the source images to the fused images. For *Q*_0_, *Q*_*W*_, *Q*_*E*_, and *Q*_*AB*/*F*_, they all lie in the interval [0,1]. For these parameters, the greater value indicates the better fusion performance. The experiments are carried out in the PC with the Intel i7-3770 CPU 3.40 GHz and 4 G RAM, operating under MATLAB R2010a.

### 3.1. The CT Images and MR Images Fusion

**Figure 5:**
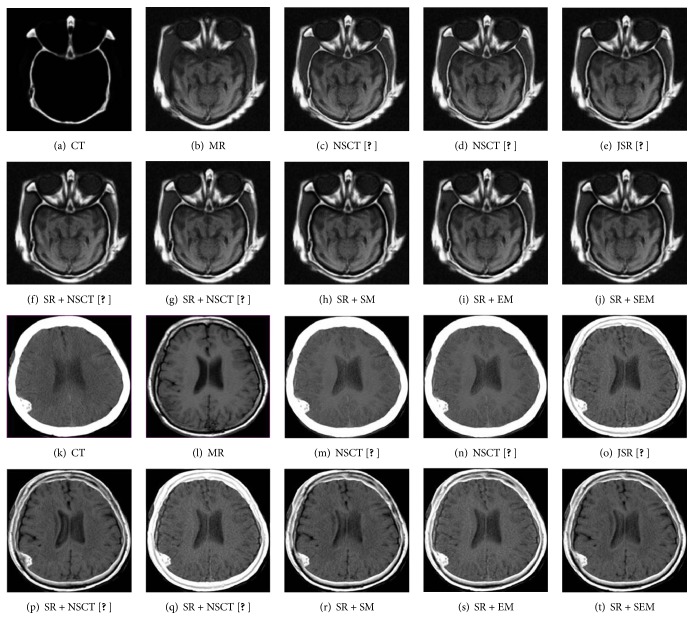
The CT and MR image fused results of different fusion methods.

In the first experiment, the CT and MR images are fused with eight different image fusion methods listed above. We used 12 groups CT and MR images to test the performance of these methods as shown in Figure 3. Two groups of results are shown in Figure 5. It is obviously seen that the results of NSCT are fuzzy in some parts, especially in Figures 5(m) and 5(n), the results of SR + NSCT [15], SR + SM, and SR + SEM can reserve better source image boundary information than the results of the other methods. And these results have no block effects, because all the methods use the sliding window strategy, in which NSCT [1] and NSCT [6] use the window with size of 3 × 3 and the others use the window with size of 8 × 8. And results of NSCT [1], NSCT [6], JSR [12], and SR + NSCT [18] are brighter than all source images, which will lead some dim information to be hidden by light information. As shown in Figures 5(m), 5(n), 5(o), and 5(q), we cannot tell the tissue information between the skull and brain. In a certain extent, the proposed method can ease these problems and meanwhile remain the merits of the SR based methods. Comparatively, the results of SR + SEM can remain better image boundary and energy information, where we can get better anatomical information from CT images and soft tissue information from MR images simultaneously. More than that, the calcified meningioma in Figure 5(f) can be distinguished from background easily in Figures 5(r), 5(s), and 5(t). As for the fused results of CT and MR images, the average scores of quantitative evaluation metrics are listed in Table 1 and the “bold” values indicate the highest values. We can see that the proposed method SR + SEM outperforms other methods in all scores. The results fused by proposed methods are all better than normal methods.

### 3.2. The MR-T1 and MR-T2 Images Fusion

**Figure 6:**
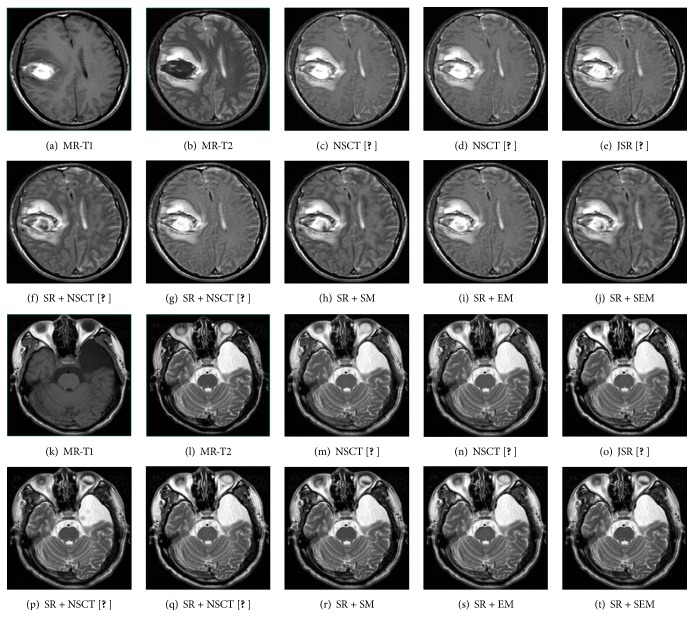
The MR-T1 and MR-T2 image fused results of different fusion methods.

In the second experiment, we used 12 groups MR-T1 and MR-T2 images to test the performance of these methods as shown in Figure 3. To illustrate the proposed fusion method, two sets of results are presented in Figure 6. In general, the results of NSCT [1], NSCT [6], and SR + NSCT [18] look gloomy and bright, demonstrating the grey distortion happens. NSCT [1], NSCT [6], JSR [12], and SR + NSCT [18] create many bad edges and make the fused results too smoothness. Comparatively, the results of SR + NSCT [15] and proposed methods show better boundary information and energy information with fewer artifacts, so that we can get better information on adipose tissue from MR-T1 images and information on vascular and tumor from MR-T2 images. Compared to the other methods, the results of proposed methods contain more information from the source images. The proposed methods preserve both better local edge and texture information, which is the vital information for diagnosis. The subacute premature hematoma is seen clearly in Figure 6(l), and we can see the location and contour of the intracranial hematoma in Figures 6(r), 6(s), and 6(t). For 12 MR-T1 and MR-T2 fused results, the average scores of quantitative evaluation metrics are listed in Table 2 and the “bold” values indicate the highest values. We can see that SR + SEM outperforms other methods in all scores. In general, the results fused by proposed methods are better than the other methods.

### 3.3. The CT Images and PET Images Fusion

**Figure 7:**
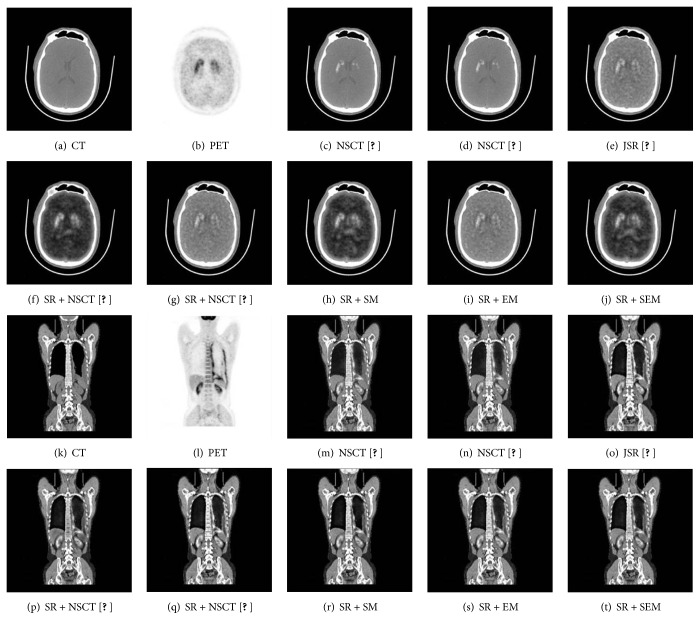
The CT and PET image fused results of different fusion methods.

In the third experiment, we used 12 CT and PET image pairs to test the performance of these methods as shown in Figure 3. Two sets of results are shown in Figure 7. Comparatively, the results of NSCT [1] and NSCT [6] are best especially in energy information, so that the fused images can capture both more spatial information in the CT images and functional information contents in PET images. However, in clinical applications, doctors need to see the position of bone and tumor to determine pathology and aid in diagnosis. The results fused by SR + SEM contain more detailed information and higher contrast but without information distortion so that we can see the outline of the kidney clearly in Figures 7(r), 7(s), and 7(t). Nasopharyngeal carcinoma can be seen in Figure 7(b), and we can use the result fused by proposed method to locate it in Figure 7 SR + NSCT [15], SR + SM, and SR + SEM easily, where are helpful to view the tumor activity, allowing physicians to better understand the effect of cancer treatment. For 12 CT and PET fused results, the average scores of quantitative evaluation metrics are listed in Table 3 and the “bold” values indicate the highest values. We can see that the SR + SEM outperforms other methods in all scores. It shows that this approach is flexible and stable.

### 3.4. The Time Complexity Analysis

**Figure 8:**
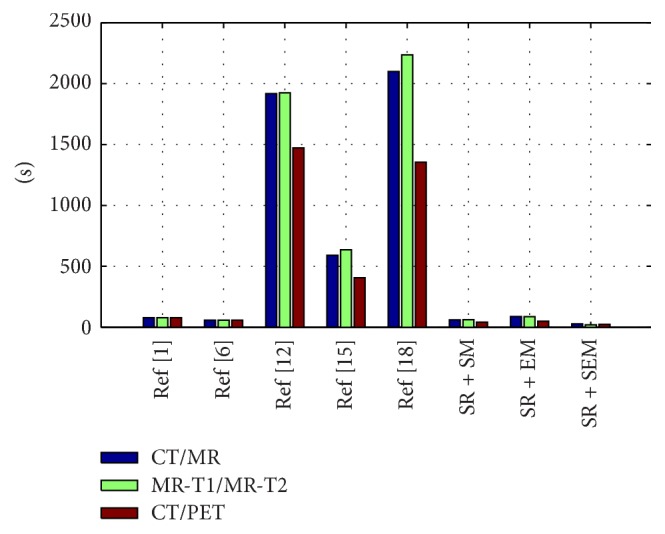
The time consuming of different fusion methods.

To realize the fusion and reconstruction of 3D medical images, a lot of CT/PET and MR/PET image slices need to be fused firstly [37, 38]. Therefore, there is a need to find a faster and stronger image fusion algorithm. As shown in Figure 8, we record the average time consumption of different methods for 36 different medical image pairs listed. It is evident that the multiscale approaches including NSCT [1] and NSCT [6] are very fast while the SR based approaches (JSR [12], SR + NSCT [15], and SR + NSCT [18]) take much more time. Comparably, the time consuming of SR + SM is about 1/20, SR + EM is about 1/20, and SR + SEM is about 1/50 of the SR based approach. From the above analysis and discussion, we draw the conclusion that SR + SEM outperforms all the others in the field of medical image fusion. Because it contains more original information from source images and better local structure information, our methods are more appropriate for doctors to localize the abnormal masses and tumors in patients.

## 4. Conclusion

In this paper, a new medical image fusion approach based on SR and feature extraction is proposed. There are at least three major improvements compared with the conventional SR based fusion methods. Firstly, we put forward three decision maps to improve quality of the SR based image fusion methods in extracting the structure and energy features of the source images. This strategy can help remain the original information from the source images as much as possible. Secondly, we add the decision map into the SR based methods to improve the speed of the algorithm. It takes only 1/50 of the time that the standard SR method needs to realize the image fusion based on proposed approach. Thirdly, adding the structure and energy information of source images into the decision map improve the quality of the fused results a lot. The experiments results indicate that the proposed fusion approach can achieve better results than the conventional fusion methods in both subjective and objective aspects.

## Figures and Tables

**Table 1:** The objective evaluation and running time for CT and MR image fused results of all methods.

	*Q*_0_	*Q*_*W*_	*Q*_*E*_	*Q*_*AB*/*F*_	MI
NSCT [1]	0.5960	0.7511	0.5707	0.5857	4.6665
NSCT [6]	0.5958	0.7554	0.5793	0.5892	4.6711
JSR [12]	0.6493	0.8148	0.6296	0.5838	3.6120
SR + NSCT [15]	0.6611	0.8477	0.6249	0.6447	4.0421
SR + NSCT [18]	0.6527	0.8161	0.5572	0.5866	3.6371
SR + SM	0.6566	0.8466	0.6992	0.6630	4.5467
SR + EM	0.6643	0.8355	0.6919	0.6477	4.2687
SR + SEM	**0.6679**	**0.8477**	**0.7043**	**0.6677**	**4.6721**

**Table 2:** The objective evaluation and running time for MR_T1 and MR_T2 image fused results of all methods.

	*Q*_0_	*Q*_*W*_	*Q*_*E*_	*Q*_*AB*/*F*_	MI
NSCT [1]	0.6311	0.8178	0.6166	0.6374	5.4548
NSCT [6]	0.6316	0.8202	0.6192	0.6387	5.4252
JSR [12]	0.6732	0.8369	0.6367	0.5744	3.9141
SR + NSCT [15]	0.6896	0.8543	0.6313	0.6608	4.8713
SR + NSCT [18]	0.6741	0.8385	0.5563	0.5774	3.9210
SR + SM	0.6832	0.8520	0.7029	0.6676	5.3846
SR + EM	0.6911	0.8542	0.6989	0.6577	4.9104
SR + SEM	**0.6991**	**0.8558**	**0.7063**	**0.6746**	**5.4563**

**Table 3:** The objective evaluation and running time for CT and PET image fused results of all methods.

	*Q*_0_	*Q*_*W*_	*Q*_*E*_	*Q*_*AB*/*F*_	MI
NSCT [1]	0.5083	0.9442	0.7297	0.7783	4.8657
NSCT [6]	0.5132	0.9475	0.7414	0.7862	4.9470
JSR [12]	0.4988	0.9590	0.8095	0.7558	3.5924
SR + NSCT [15]	0.4983	0.9635	0.7628	0.7863	4.0568
SR + NSCT [18]	0.5046	0.9612	0.7564	0.7625	3.6635
SR + SM	0.4992	**0.9652**	0.8337	0.8030	4.7986
SR + EM	**0.5234**	0.9642	0.8311	0.7984	4.5778
SR + SEM	0.5100	**0.9652**	**0.8369**	**0.8041**	**4.9720**
